# Metabolic Concepts in Idiopathic Intracranial Hypertension and Their Potential for Therapeutic Intervention

**DOI:** 10.1097/WNO.0000000000000684

**Published:** 2018-07-06

**Authors:** Catherine Hornby, Susan P. Mollan, Hannah Botfield, Michael W. O'Reilly, Alexandra J. Sinclair

**Affiliations:** Department of Metabolic Neurology (CH, SPM, HB, AJS), Institute of Metabolism and Systems Research, College of Medical and Dental Sciences, University of Birmingham, Birmingham, United Kingdom; Birmingham Neuro-Ophthalmology Unit (SPM, AJS), Ophthalmology Department, University Hospitals Birmingham NHS Foundation Trust, Birmingham, United Kingdom; Centre for Endocrinology, Diabetes and Metabolism (HB, MWOR, AJS), Birmingham Health Partners, United Kingdom; and Department of Neurology (AJS), University Hospitals Birmingham NHS Foundation Trust, Birmingham, United Kingdom.

## Abstract

**Background:**

Traditional risk factors associated with idiopathic intracranial hypertension (IIH) include obesity, weight gain, and female sex. The incidence of IIH is increasing and yet the underlying trigger and the fueling pathological mechanisms are still poorly understood.

**Evidence Acquisition:**

Review of ophthalmology, neurology, general surgery, obesity, endocrinology, nutrition, and neurosurgery literature was made.

**Results:**

The facts that implicate sex and obesity in IIH and headache are examined. The role of fat distribution in IIH is questioned, and the concept of adipose tissue functioning as an endocrine organ driving IIH is discussed. The impact of androgen metabolism in IIH is reviewed as is the emerging role of glucagon-like-peptide-1 analogues in modulating intracranial pressure. This introduces the concept of developing targeted disease-modifying therapeutic strategies for IIH.

**Conclusions:**

This review will discuss the possible role of the adipose/gut/brain metabolism axis in IIH and speculate how this may impact the pathogenesis of IIH and therapeutic opportunities.

Idiopathic intracranial hypertension (IIH) is a condition of increased intracranial pressure (ICP) of unknown etiology (1). Clinicians often debate the principal pathogenic mechanisms, but no unifying theory has yet emerged. Cerebrospinal fluid (CSF) dynamics (overproduction or underdrainage) is likely a result of an initial trigger or ongoing insult. Cerebral venous sinus stenosis may occur in IIH, although its role in the disease pathogenesis is debated. Exploration of the well-known associations of female sex, obesity, weight gain, and polycystic ovary syndrome (PCOS) has led to the active investigation of the role of adipose tissue, androgens, and gut peptides in modulating ICP in IIH. A pathological basis for IIH, which may also contribute to headache in IIH, could potentially involve how the gut/brain metabolism axis interacts. For IIH, these newly described pathways could lead to targeted weight loss therapies, such as customized bariatric surgery, or other novel therapeutics such as glucagon-like-peptide-1 (GLP-1) receptor agonists. This review will discuss the possible role of the adipose/gut/brain metabolism axis in IIH and speculate how this may impact pathogenesis and therapeutic opportunities.

## THE EVIDENCE IMPLICATING SEX AND OBESITY

IIH predominately affects women, with less than 10% of affected patients being men (1–4). There also is an association between IIH and obesity, as over 90% of patients are obese (5). The incidence of IIH is rising and seems to be increasing with the general trend of rising obesity rates (6), with the age- and sex-adjusted annual incidence more than doubling in the period between 2002 and 2014 compared with 1990–2001 in 1 study in the United States increasing from 1.0 to 2.8 per 100,000 (7). When stratified for reproductive age, female sex, and weight, the incidence has been reported between 12 and 28/100,000 per year (8). The risk of IIH also increases with increasing body mass index (BMI) (8). The greater degree of weight gain the year before symptom onset results in a greater risk of IIH. Weight loss has been shown to result in clinical improvement in several parameters, including reduction in ICP, papilledema, and headache (9). However, for an individual patient, the amount of weight modification needed to put the disease into remission varies, which raises a question of whether it is the type of adipose tissue or its location that is more relevant than the absolute amount of adipose tissue that is lost.

## QUESTIONING THE ROLE OF FAT DISTRIBUTION

There is a strong association between abdominal fat deposition and health outcomes, including an increased risk of type 2 diabetes, adverse cardiovascular effects, insulin resistance, and dyslipidemia (10–12). The pattern of fat distribution in patients with IIH may be of relevance. One long-standing theory is that the mechanical effects of excessive abdominal fat elevate intra-abdominal pressure, which increases intrathoracic pressure and, thereby, increases cerebral venous pressure, and, consequently ICP (13). However, this hypothesis would not explain why only a proportion of all individuals with obesity develop IIH. Some studies measuring waist–hip ratios have reported increased lower-body fat in IIH as opposed to centripetal obesity (4,14). The use of dual-energy x-ray absorptiometry scanning, a validated accurate method to define and quantify body fat deposition, revealed that patients with IIH have a similar, centripetal fat distribution compared with simple obesity (15). Interestingly, in IIH, truncal fat mass correlates with opening pressure on lumbar puncture although BMI does not. However, we cannot exclude a false negative error affecting these results (15). It has also been noted that fat is preferentially lost from the trunk compared with the limbs in patients with IIH undergoing therapeutic weight loss resulting in significant reduction of ICP. This potentially implies that truncal fat mass may be pathogenic in IIH. It is also interesting to note that although BMI does not correlate with LP pressure in patients with IIH, there is a significant correlation between BMI and LP pressure in patients who do not have increased ICP disorders (16).

## EARLY INDICATIONS THAT ADIPOSE TISSUE IS THE ENDOCRINE ORGAN DRIVING INTRACRANIAL HYPERTENSION

Adipose tissue functions as an endocrine organ, secreting a myriad of factors including proinflammatory cytokines, chemokines, adipokines, and hormones (17) that may be involved in IIH pathogenesis. Of note, some reports have indicated that the adipokine, leptin, is elevated in the serum (18) and CSF of patients with IIH, independent of BMI, compared with BMI-matched control subjects (19). There was a correlation of leptin levels between CSF and serum in patients with IIH. In addition, the increased CSF–serum leptin ratios in patients with IIH compared with controls suggest that transfer of leptin over the blood–brain barrier is not impaired in IIH, in contrast to obesity. Leptin is involved in hypothalamic control of satiety and weight regulation (20), and typically low CSF leptin levels are found in patients with obesity. It is, therefore, curious that CSF leptin levels are high in IIH (consequently driving hypothalamic satiety), yet those patients remain obese. This has increased the possibility of hypothalamic leptin resistance in IIH. Leptin receptors are localized to the choroid plexus and contribute to leptin transport in to the CSF (21). In addition, choroid plexus is the principle site for CSF secretion. However, the inconsistent findings from research in this area diminish the likelihood that leptin has a dominant role in pathogenesis, but aberrant leptin signaling may be a contributing factor for obesity in IIH (19).

Obesity is a chronic inflammatory condition and, consequently, another theory is the possibility of pathogenic inflammation causing IIH (22–24). Investigation of the cytokine and chemokine profiles in both the serum and CSF of patients with IIH has shown a significantly increased level of chemokine profiles in both the serum and CSF of patients with IIH has shown a significantly increased level of the chemokine CCL2 in the CSF of patients with IIH compared with controls, whereas CCL8, CCL7, and IL-1α were increased in the plasma in IIH, but not significantly (25). However, this study did not use a control group that was well matched to the patients with IIH as BMI of the IIH cohort was significantly higher than that of controls. Therefore, these findings could be representative of the inflammatory profile in obesity rather than in IIH. In addition, there were no significant differences in the levels of serum and CSF interleukin-8 (IL-8), monocyte chemoattractant protein-1 (MCP-1), resistin plasminogen activator inhibitor-1 (PAI-1), tissue necrosis factor-α (TNF-α), and hepatocyte growth factor (25). CSF IL-6 was also not significantly different in patients with IIH nor were CSF:serum cytokine ratios. However, others have found a proinflammatory cytokine profile in patients with IIH, with significant elevations in IL-2 and IL-17 in the CSF of patients with IIH compared with controls with other neurological conditions. There was no difference in serum cytokine levels, but a potential confounder was that the controls were not BMI matched with the patients with IIH (26).

Obesity occurs in conditions with excess secretion of glucocorticoids such as Cushing syndrome. Systemic levels of cortisol are regulated by the hypothalamic–pituitary–adrenal axis. At a tissue level, cortisol is regulated by the enzyme 11β-hydroxysteroid dehydrogenase (11β-HSD). 11β-HSD type 1 (11β-HSD1) which converts inactive cortisone into cortisol and is found in high levels in the liver and adipose tissue (27). Mice overexpressing 11β-HSD1 globally, or specifically in adipose tissue, have a metabolic syndrome phenotype of obesity, dyslipidemia, and glucose intolerance (28). In humans, obesity is associated with normal systemic cortisol levels but dysregulated 11β-HSD1 activity in adipose tissue (29). The relationship between glucocorticoids and IIH is somewhat more complicated, as IIH may occur during withdrawal or replacement of glucocorticoid therapy (30). Activity and expression of 11β-HSD1 is especially high in fat and, interestingly, 11β-HSD1 has been localized to the choroid plexus, where it drives local cortisol production (31). Cortisol generation in the choroid plexus is believed to drive epithelial sodium transporters on the apical membrane of the choroid plexus epithelial cells and establish an ionic gradient, promoting CSF production. 11β-HSD1 may, therefore, have a key role in influencing the regulation of CSF secretion, and thus ICP. Preliminary studies in patients with IIH have demonstrated that therapeutic weight loss causes a reduction in global 11β-HSD1 activity, which correlates with the reduction in ICP (32). The effects of 11 β-HSD1 inhibition in reducing ICP in patients with IIH is currently being assessed in a randomized clinical trial (33).

## POSTULATING THE ROLE OF ANDROGEN METABOLISM IN INTRACRANIAL HYPERTENSION

Polycystic ovary syndrome (PCOS) and IIH share many overlapping clinical features. PCOS is the most common endocrine condition in women of reproductive age, and androgen excess is a cardinal defining feature in most diagnostic consensus criteria (34,35). The most severe clinical and biochemical features of androgen excess in PCOS are observed in the context of comorbid obesity (36). A number of reports have found that the prevalence of PCOS is significantly higher in women with IIH women compared with the general female population (up to 57% compared with 5%–10%). However, these studies did not compare cohorts matched for age and BMI and, consequently, this could represent a chance association (37). It is possible that obesity, often noted in women with PCOS, could represent the pathogenic driver in those individuals with coexisting IIH and PCOS. However, although PCOS is more common in women with obesity, it can also occur in women of normal body weight, so the association of IIH and PCOS may not be due to obesity alone. Of interest, the prevalence of PCOS is reported as significantly lower in female cohorts with simple obesity (28.3% in 1 study) compared with the incidence reported in patients with IIH (57%) (38).

It has been proposed that androgen excess may be a risk factor in women with IIH, independent of obesity, and that hyperandrogenism may play a pathophysiological role in disease onset. One study found that androgen excess was associated with a younger age at onset of IIH, but did not correlate with BMI, waist-to-hip ratios, or duration of IIH (39). Patients with IIH have increased adipose tissue, and androgen activation in adipose tissue may be relevant to the pathophysiology of IIH. Adipose tissue plays a major role in peripheral androgen generation (40), and increased intra-adipose androgen activation is observed even in women with simple obesity (41).

Case reports of transgender patients undergoing female to male gender reassignment provide important insights into the role of androgens in IIH. Five case reports detail that patients receiving testosterone injections subsequently developed IIH, 4 of whom showed a temporal relationship between commencement of testosterone and symptoms of IIH (Table 1) (42–46). There needs to be caution when interpreting these reports because confounders such as weight gain may have influenced the development of increased ICP. Further evaluation of the role of testosterone in ICP regulation would be of great interest.

**TABLE 1.**
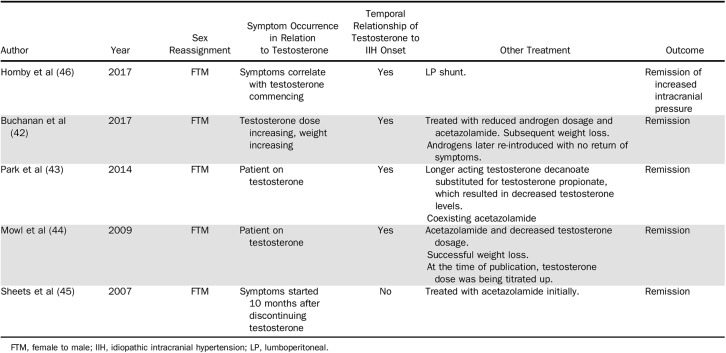
Reports of idiopathic intracranial hypertension in patients with sex reassignment

IIH in men, although uncommon, also provides insights into the complex and potentially sexually dimorphic role that androgens may play in this disorder. Men with androgen deficiency may be at increased risk of IIH; case reports of developing IIH after induction of hypogonadism with androgen deprivation therapy for prostate cancer have been reported (47). It also has been documented that men with IIH are more likely to have symptoms of testosterone deficiency, such as erectile dysfunction and reduced libido (48).

We suggest that a pathophysiological window of abnormal circulating androgen levels, with serum testosterone concentrations at a level shared by men with androgen deficiency and women with androgen excess, could increase the risk of IIH. Sexually dimorphic associations of androgens are well recognized. Metabolic diseases such as nonalcoholic fatty liver disease and diabetes are noted to have a sexually dimorphic relationship to androgens (49). In these metabolic conditions, women with androgen excess and men with androgen deficiency have an overlapping constellation of adverse cardio-metabolic risk factors (50). Circulating testosterone concentrations in women with androgen excess may overlap with those of men with androgen deficiency; at these serum concentrations, there is preferential accumulation of visceral adiposity in both sexes and metabolically deleterious loss of skeletal muscle bulk in men (51).

Therefore, it is conceivable that IIH represents a distinct neurological manifestation of this adverse metabolic phenotype with female androgen excess and male androgen deficiency conferring a risk factor for the development of IIH. Although female androgen excess and male hypogonadism are associated with abdominal obesity, which could be the trigger for IIH in these cases, it is also important to note that severe hypogonadism, with low androgen levels, can occur independently of obesity in men and remains associated with increased cardiovascular and all causes of mortality (52). Equally, women with PCOS and androgen excess are at increased risk of metabolic complications, such as nonalcoholic fatty liver disease, independent of obesity (53). Further studies in men and women are needed to understand these complex sexually dimorphic associations and their relationship to IIH and ICP regulation in the context of obesity.

There also is very limited understanding of the roles of estrogen and progesterone in IIH (54,55). Studies to date describing estrogen (estrone) and progesterone levels in the CSF have been small (n = 7 and n = 15), lacking matched control groups, and hampered through the use radioimmunoassays with a poor lower limit of detection. This has led to levels of estrogens being inconsistently detected in the CSF. Estrogen receptors are expressed in the choroid plexus epithelial cells, however, and their potential involvement in CSF secretion is an interesting avenue for future studies (56,57).

### The Emerging Role of Gut Peptides in Modulating Intracranial Pressure

Glucagon-like peptide-1 (GLP-1) is a gut peptide secreted by the distal small intestine in response to food intake and synthesized in neurons of the solitary tract nucleus. GLP-1 stimulates glucose-dependent insulin secretion and inhibits glucagon release, lowering blood glucose (58) and promoting satiety and weight loss (59). GLP-1 mimetics are currently being used clinically to treat obesity and diabetes. In addition, GLP-1 has effects on renal proximal tubule sodium ion (Na^+^) secretion by reducing Na^+^ resorption into the blood stream and increasing diuresis (60). In the brain, CSF secretion by the choroid plexus epithelial cells is also driven by the net movement of Na^+^ from the blood into the cerebral ventricles. The localization of the receptor to the choroid plexus (61,62) suggests that GLP-1 may be able to modulate Na^+^ transport in the choroid plexus and, thereby, CSF production. Recently, it was demonstrated that exendin-4, which is a GLP-1 receptor agonist, also known as exenatide, currently prescribed to treat obesity and diabetes, reduces Na+/K+ATPase activity a marker of CSF secretion. Of note, exendin-4 dramatically reduced ICP in rodents with increased ICP. Exendin-4 reduced ICP by 44% within 10 minutes of dosing. Not only did the treatment effects last for at least 24 hours, cumulatively, the doses lowered the baseline ICP (61). Although there is no current evidence that gut neuropeptides or GLP-1 are implicated in the pathogenesis of IIH, these studies do highlight the therapeutic potential for GLP-1 receptor agonist in the management of IIH.

## DEVELOPING TARGETED DISEASE-MODIFYING STRATEGIES FOR INTRACRANIAL HYPERTENSION

Weight loss typically results in clinical resolution of IIH (9,63,64). A large number of case reports discuss the use of bariatric surgery as a treatment for IIH, reporting significant improvement of symptoms and clinical signs in most patients (65–71). The IIH weight study (IIH:WT) (72) finished recruitment in April 2017 and will test the hypothesis that bariatric surgery will be more beneficial in the long term for treating IIH compared with community weight loss programs.

There are several bariatric surgical approaches, including Roux-en-Y gastric bypass, laparoscopic adjustable gastric banding, and laparoscopic sleeve gastrectomy. All 3 surgical methods result in a greater degree of weight loss compared with nonsurgical interventions (73). Reports of the effect of bariatric surgery on outcomes such as diabetes, lipid profile, and blood pressure are variable. One study found remission of type 2 diabetes in 28.6%–67.5% of patients after surgery (depending on the procedure) (74). What is relevant is that gastric bypass, in particular Roux-en-Y bypass, enhances GLP-1 levels contributing to the beneficial effects of bariatric surgery in diabetes (75). Given the emerging role of gut peptides in modifying ICP, it is interesting to speculate that bariatric surgery techniques that modify GLP-1 to the greatest degree (Roux-en-Y bypass), may be most effective in inducing IIH remission, not only by virtue of weight lost but also because of the effect on gut peptides and potentially altering in CSF secretion.

**FIG. 1.**
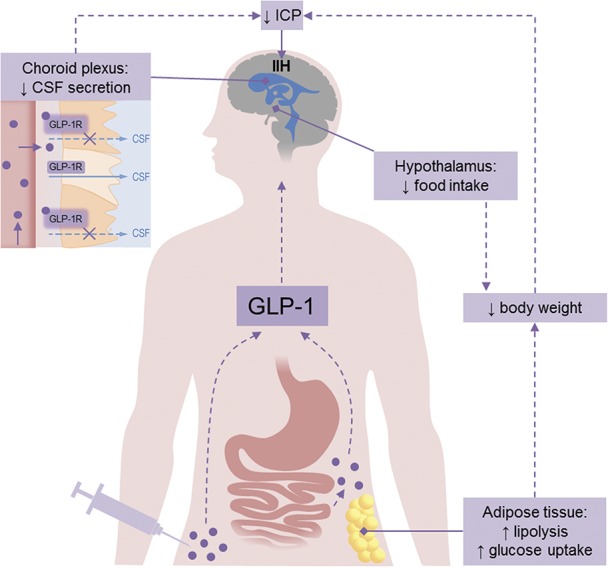
Glucagon-like peptide 1 (GLP-1) mechanism of action in IIH. Normally GLP-1 (purple circles) is produced by L-cells in the small intestine in response to food. GLP-1 mimetics are used to treat diabetes and obesity and are administered by subcutaneous injection. GLP-1 mimetics could be beneficial for patients with IIH as they act on the hypothalamus to reduce food intake and on adipose tissue to increase lipolysis resulting in weight loss. In addition, GLP-1 mimetics bind to GLP-1 receptors (GLP-1R) on the choroid plexus, leading to a reduction in CSF secretion and ICP. CSF, cerebrospinal fluid; ICP, intracranial pressure; IIH, idiopathic intracranial hypertension.

Future therapies would ideally modify CSF secretion to control ICP but also induce weight loss. The novel data on the properties of GLP-1 receptor agonist to reduce ICP (61) may represent an important advance that would have these properties. Exenatide, a synthetic version of exendin-4, a GLP-1 receptor agonist, is widely used to treat diabetes and obesity and has the potential to be repurposed for the treatment of increased ICP (Fig. 1). Furthermore, exenatide has received orphan drug designation for the treatment of IIH from the European Medicines Agency and the US Food and Drug Administration. A clinical trial is currently underway exploring the physiological effects of exenatide in reducing ICP in a small cohort of active patients with IIH.

## CONCLUSIONS

**FIG. 2.**
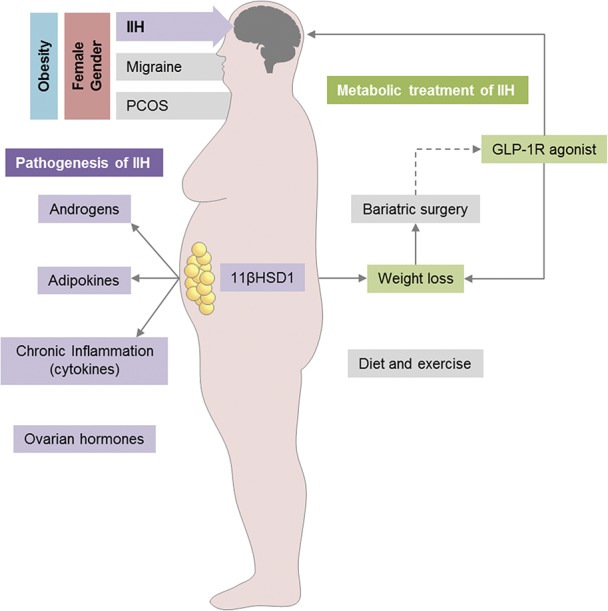
Metabolic pathogenesis and treatment in IIH. IIH is most prevalent in obese women of childbearing age, similar to migraine and polycystic ovary syndrome. A combination of dysregulated adipose tissue and hormones may be involved in IIH pathogenesis. Weight loss through bariatric surgery and diet has been shown to improve IIH symptoms. GLP-1R agonists may have additional benefits for patients with IIH by directly affecting ICP and causing weight loss. GLP-1R, glucagon-like-peptide-1 receptor; ICP, intracranial pressure; IIH, idiopathic intracranial hypertension.

The emerging link between IIH and metabolism is potentially important. The areas outlined in this review start to establish a link between IIH and the concept of metabolic dysregulation (Fig. 2).

The underlying pathophysiological mechanism of IIH has yet to be established. However, the answer may lie in the location and metabolic activity of the excessive adipose tissue in these patients. Ongoing randomized controlled trials will potentially provide data that will help uncover the relationship between obesity, weight loss, and IIH (72). Novel, in-vivo and in vitro data suggest that exenatide, a GLP-1 receptor agonist, may represent an ideal therapy for IIH through rapid reduction of CSF secretion at the choroid plexus, lowering ICP in addition to its weight loss properties. Expanding our knowledge of the metabolic pathways involved in ICP regulation and IIH maybe an important step for future research, which could lead to the identification of novel targeted therapies for patients with IIH.

STATEMENT OF AUTHORSHIP

Category 1: a. Conception and design: C. Hornby, S. P. Mollan, and A. J. Sinclair; b. Acquisition of data: C. Hornby, S. P. Mollan, H. Botfield, M. W. O'Reilly, and A. J. Sinclair; c. Analysis and interpretation of data: C. Hornby, S. P. Mollan, H. Botfield, M. W. O'Reilly, and A. J. Sinclair. Category 2: a. Drafting the manuscript: C. Hornby, S. P. Mollan, H. Botfield, M. W. O'Reilly, and A. J. Sinclair; b. Revising it for intellectual content: C. Hornby, S. P. Mollan, H. Botfield, M. W. O'Reilly, and A. J. Sinclair. Category 3: a. Final approval of the completed manuscript: C. Hornby, S. P. Mollan, H. Botfield, M. W. O'Reilly, and A. J. Sinclair.
